# Celiac Artery Thrombosis and Superior Mesenteric Artery Stenoses with Essential Thrombocythemia: A Case Report

**DOI:** 10.1155/2012/741653

**Published:** 2012-12-11

**Authors:** Hasan Attila Keskin, Fahri Yetisir, Huseyin Bayram, Mehmet Selahattin Bayraktaroglu, Erdal Simsek, Mehmet Kilic, Salih Fehmi Katircioglu

**Affiliations:** ^1^Department of Cardiovascular Surgery, Etlik Education and Research Hospital, 06020 Ankara, Turkey; ^2^Department of General Surgery, Etlik Education and Research Hospital, 06020 Ankara, Turkey

## Abstract

Thrombosis of the celiac artery trunk is a rare cause of acute abdominal pain. Thrombosis of the celiac artery carries a high mortality and morbidity when the diagnoses and treatment are delayed. It is frequently associated with other cardiovascular events. The most common etiology is atherosclerosis. 20–30% of cases may have symptoms of chronic mesenteric ischemia. Main goal of the treatment is to reestablish the diminished or stopped mesenteric blood flow and to avoid end-organ ischemia. Essential thrombocythemia is a chronic myeloproliferative disorder characterized by marked increase in thrombocyte number and clinical presentation may be with thrombotic episodes, hemorrhage, or both. To our knowledge this is the first report of celiac artery thrombosis and superior mesenteric artery stenoses in a patient with essential thrombocythemia. The patient was managed successfully with surgical treatment.

## 1. Introduction

Thrombosis of the celiac artery trunk is a rare cause of acute abdominal pain. Conditions that increase the tendency towards thrombosis like atherosclerosis, collagen tissue disorders, coagulation abnormalities, and malignancies can be noted among the leading causes of celiac artery thrombosis. Angiography is the gold standard of the diagnoses. The purpose of treatment is to reestablish blood flow in the mesenteric vessels and to prevent end-organ ischemic damage and infarct. Percutaneous angioplasty and surgical treatment are the preferred methods of treatment. Celiac artery thrombosis is usually associated with other cardiovascular diseases. Although there is substantial development in diagnoses and treatment of celiac artery thrombosis, hospital mortality is still rated at 59–93%. Successful treatment depends on early diagnoses and effective intervention either surgically or endovascularly to reestablish blood flow and surgical resection of necrotic parts and good intensive care unit management [1].

Essential thrombocythemia (ET) is a myeloproliferative disorder characterized by significant increase in thrombocyte number. Clinically it can present as either hemorrhage or thrombotic episodes or both of them [2].

In this report we presented a case who had ET with superior mesenteric artery (SMA) stenoses associated with celiac artery thrombosis.

## 2. Case Report

**Figure 1:**
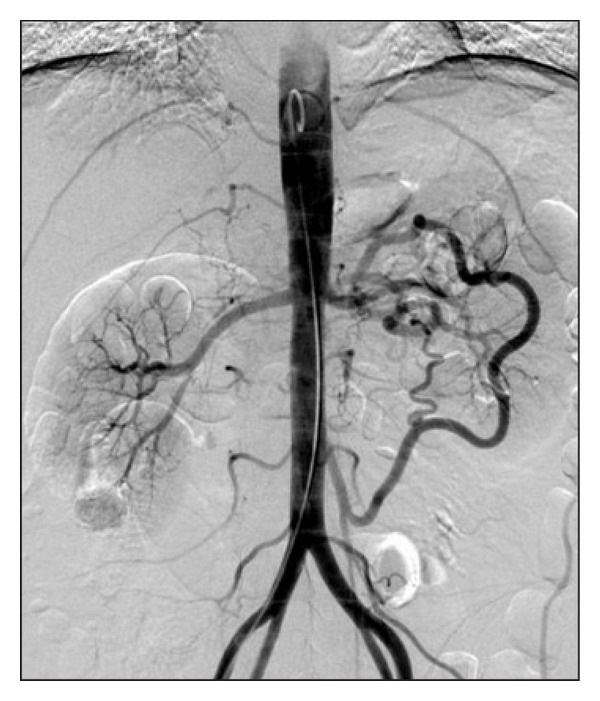
Preoperative DSA imaging.

A 44-year-old male patient with sudden onset of abdominal pain around the navel, nausea, and vomiting reported to the emergency unit, and splenic infarction was observed in the abdominal ultrasonography before 1-month admission to our clinic. A complete blood count demonstrated a thrombocyte count of 1150000/mm^3^, and splenectomy was performed in this centre. Hemorrhagic infarct with vascular thrombosis was detected in the pathologic examination of the spleen. Due to unrelenting abdominal pain he was referred to a higher centre where a contrast-enhanced abdominal CT was performed, and a thrombus was identified in the celiac artery trunk. There were no other risk factors of thrombosis except for smoking in his medical history. Widespread tenderness of the abdomen but no peritoneal irritation findings like rebound or defence was detected on physical examination in our clinic. Blood pressure was 90/60 mmHg, pulse rate was 84 beats/minute, and body temperature was 36.9°C. There was no evidence of abnormality apart from lowered total protein and albumin values (6.2 mgr/dL and 3.2 mgr/dL, resp.) on biochemical samples. Complete blood count was as follows: hemoglobin: 11.9 g/dL, white blood cells: 11300/mm^3^, and thrombocyte: 781000/mm^3^. Prothrombin time was slightly elevated (International Normalized Ratio: 1.55). Patient was anticoagulated with low molecular weight heparin. Enteral nutritional feeding was poor, so total parenteral nutrition was initiated through a central venous line. Digital subtraction angiography (DSA) revealed obstructed celiac artery and severe stenoses at the origin of superior mesenteric artery. DSA also revealed that the superior mesenteric artery was filled in a retrograde direction through the riolan arc through the inferior mesenteric artery. The inferior pancreaticoduedonal artery filled from the proximal segment of SMA and the hepatic artery was therefore poorly visualized (Figure 1). 

**Figure 2:**
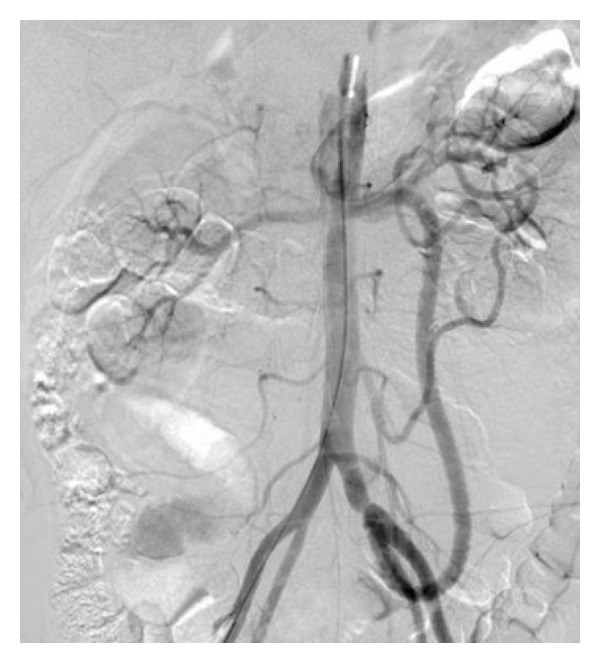
Postoperative 2. month control DSA imaging.

Patient was operated under these conditions, and a bypass graft from left common iliac artery to superior mesenteric artery was performed using an 8 mm heparin-coated expanded polytetrafluorethylene graft (Figure 2). Thrombectomy to celiac artery was not performed because of the atherosclerotic origin of SMA. Also atherosclerosis in celiac artery trunk was considered initially. Aortotomy was not performed in this case.

Symptoms subsided quickly after the operation. Oral intake was started on postoperative day 5 without any complaints. The patient was discharged from hospital in good condition, and a fully patent bypass graft was observed on a follow-up check DSA. The patient was referred to the hematology unit for follow-up of thrombocytosis. Protein C and protein S levels were studied. There was no lack of protein levels. Bone marrow biopsy was performed, and the findings in favor of ET (myeloproliferative neoplasia, rate of cellularity: 60%, myeloid/erythroid ratio: 3/1, and increase in number of megakaryocytes). Chromosomal analysis was performed. Jak2 (v617f) gene mutation was detected. Finally anagrelide therapy was started.

## 3. Discussion

Although acute mesenteric ischemia is a rarely observed abdominal emergency, it has a high mortality rate. Atherosclerosis, Behcet disease, thrombocytosis, protein S, protein C, antithrombin III deficiency, and malignancies which increase thrombotic tendency can be counted among the major etiologic factors of acute mesenteric ischemia [1]. Patients may present with sudden onset abdominal pain or nonspecific abdominal complaints. Direct abdominal X-ray and ultrasonography are helpful in diagnoses, but angiography is the primary diagnostic method. Angiography is also helpful in the exact localisation of the obstruction and the condition of collateral circulation. In our case the first symptom of ET was acute abdominal pain due to celiac artery thrombosis. It is important to emphasize that the first finding of ET may be thrombosis.

Early diagnoses and treatment of acute mesenteric ischemia are apparently demonstrated in many reports. Johnston et al. [3] performed mesenteric arterial bypass in 34 patients with acute or chronic mesenteric ischemia, and they reported good outcomes in both early and late results.

C. W. Kim and J. W. Kim [4] reported a case of celiac artery thrombosis, and splenic infarction was surgically treated, and it was related to protein S deficiency. In our case, a disease with predisposition to thrombocytosis was diagnosed as ET. There was thrombosis of the celiac trunks along with SMA stenosis, which were treated successfully surgically, and there was no lack of protein C, S levels.

ET is a myeloproliferative disease which is characterized by an elevation of thrombocyte number of greater than 600000/mm^3^ determined by a polycythemia vera study group. The prevalence in the general population is approximately 30/100 000. Some patients with ET are asymptomatic, others may present vasomotor, thrombotic, or hemorrhagic disturbances. Vascular occlusive events are related to cerebrovascular, coronary, and peripheral arterial circulation. The major cause of mortality is thrombosis of the large arteries which can result in neurological disabilities, cardiac events, and peripheral arterial disabilities. Therapeutic interventions in ET are limited to decisions concerning the introduction of antiaggregation therapy and/or starting thrombocyte cytoreduction. The therapeutic value of hydroxycarbamide and aspirin in high-risk patients has been supported by controlled studies [2]. In our case there were no other reasons identified as a possible cause of thrombocytosis like infectious diseases or other myeloproliferative disorders. The bone marrow biopsy supported the diagnoses of ET. 

In case of suspicion of acute mesenteric ischemia, emergency angiographic imaging and immediate revascularization are life saving. Endovascular balloon angioplasty and/or surgical bypass are the preferred treatment options. Endovascular interventions are recently becoming more popular due to the lower complication rates in contrast to surgical treatment. A report comparing the endovascular and surgical treatment of mesenteric ischemic patients demonstrated that early inhospital complication rates were higher in the surgically treated group, whereas a 3-year mortality rate was similar. However, after 3 years the symptomatic recurrence rate was significantly higher in the angioplasty group [5]. Biebl et al. [6] also reported similar results stating that the surgical group has better long-term results with less need of secondary interventions. 

Mesenteric bypass surgery is a treatment option with better long-term results for mesenteric ischemia. In these operations, the aorta or iliac arteries could be preferred as inflow vessels. Antegrade bypass may be better for patency. However, when aorta-mesenteric bypass is the treatment of choice, aorta has to be clamped temporarily to perform aortic inflow anastomosis. Clamping of the aorta might be troublesome because of highly possible associating thrombus and intense atherosclerosis of the aorta. Therefore, according to our opinion, providing mesenteric blood flow from common iliac artery is a safer method.

## 4. Conclusion

We thought that the common iliac artery may be a safer option to choose as the inflow vessel for this patient.
